# Avatar Customization and Embodiment in Virtual Reality Self-Compassion Therapy for Depressive Symptoms: Three-Part Mixed Methods Study

**DOI:** 10.2196/71004

**Published:** 2025-10-02

**Authors:** Thomas C Elliott, Yanzhuo Yang, Jarrod Knibbe, Julie D Henry, Nilufar Baghaei

**Affiliations:** 1 School of Electrical Engineering and Computer Science, Faculty of Engineering, Architecture and Information Technology, The University of Queensland, General Purpose South (78), St Lucia QLD 4067 Level 4, Brisbane, 4067, Australia, 61 0733652097; 2 School of Psychology, Faculty of Health and Behavioural Sciences, The University of Queensland, Brisbane, Australia

**Keywords:** avatar design, virtual reality, self-compassion, depression, mental health, user experience, uncanny valley

## Abstract

**Background:**

As virtual reality technologies become more accessible, understanding how design features influence user experience (UX) and psychological benefit is critical, particularly for emotionally sensitive interventions. Thus, while prior studies support the use of self-compassion paradigms in immersive virtual reality (VR) environments, the effects of avatar stylization, customization, and mirrored self-representation on therapeutic outcomes are not well understood. For instance, while it is often assumed that increasingly realistic avatars are preferable to less realistic ones, this basic premise remains largely untested.

**Objective:**

This study aimed to evaluate whether avatar appearance, customization features, and virtual mirrors affect UX and therapeutic outcomes in VR self-compassion therapy. Specifically, we examined whether stylized avatars, avatar customization, and virtual mirror feedback influenced user-rated self-compassion and depression symptoms.

**Methods:**

Across three between-subjects studies (N=107 neurotypical adults), participants engaged in an immersive individualized VR therapy protocol based on a 2-phase compassion task. The conditions were (1) stylized avatars (n=20), (2) stylized customizable avatars (n=49), and (3) stylized customizable avatars with a virtual mirror (n=38). Participants completed the User Experience Questionnaire, the Self-Compassion Scale, and the 8-item Patient Health Questionnaire (PHQ-8). In study 3, presence was also assessed using the Slater-Usoh-Steed scale. Qualitative feedback was analyzed thematically. Between- and within-study comparisons used *t* tests and Mann-Whitney *U* tests.

**Results:**

Avatar customization (study 2) led to a significant increase in self-compassion (Self-Compassion Scale: baseline mean 3.05, SD 0.98; follow-up mean 3.55, SD 1.16; *t*_89_=2.22; *P*=.03; *d*=–0.47), though PHQ-8 scores remained unchanged. The virtual mirror condition (study 3) significantly improved depression scores (PHQ-8: *U*=477.5; *z*=2.53; *P*=.01; *r*=0.30) and UX across four User Experience Questionnaire categories, including attractiveness and dependability. However, self-compassion did not significantly change in study 3 (mean 3.88, SD 1.33 → mean 4.09, SD 1.05; *t*_63_=0.71; *P*=.47; *d*=0.18). Presence scores in study 3 (mean 4.56, SD 1.58) were also comparable to real-world benchmarks. Qualitative feedback highlighted strong engagement with avatars and mirrors, and participants reported emotional safety and personalization benefits.

**Conclusions:**

Stylized avatars, when paired with customization and mirrored embodiment, can support UX and therapeutic benefit in VR self-compassion therapy. These findings challenge the assumption that hyperrealistic avatars are superior and highlight the importance of emotionally congruent design choices. The combination of stylization, individualization, and visual feedback may offer a low-barrier, user-aligned strategy for future therapeutic VR applications.

## Introduction

Virtual reality (VR) is emerging as a promising tool in therapeutic interventions for depression [1,2]. One approach involves users embodying avatars to give and receive compassion, which has been shown to lead to positive self-compassion outcomes. Customization of avatars and environments may improve these effects [3], yet the impact of such design decisions has not been systematically tested. Consequently, their influence on therapeutic outcomes remains unclear.

Much is already known about embodiment-related constructs relevant to VR therapy, including body ownership, agency, presence, and immersion [4-11]. These concepts provided were important theoretical grounding in support of a similar VR therapy study [12] and are therefore reviewed here to provide theoretical grounding in the context of the present study. Embodiment has been defined as the sense that the properties of a virtual body are experienced as one’s own [13]. Supporting conditions for agency, self-location, and ownership contribute to embodiment and can be facilitated by a first-person perspective and visuomotor synchrony.

Presence is critical to this study, enabling behavioral engagement in VR. It depends in part on embodiment—specifically on recognizing a virtual body as one’s own [14]. Immersion, however, refers to the technical capacity of the system to produce realistic and interactive environments [11]. To support immersion, this study used the Meta Quest 2 head-mounted display to support high immersion through multisensory stimulation [15].

Beyond these foundational elements, this study is situated within the domain of individualized VR (iVR), where VR experiences are tailored to the user—often through customization options. iVR has been shown to improve both user experience (UX) and therapeutic outcomes [2,3]. Customization of VR features such as adjusting game difficulty, audio, and objects from virtual environments has also been beneficial for the user [16-19]. Within iVR, avatar customization is one of many possibilities that allow users to modify the avatar’s face, body, and clothing. Prior studies have shown that customization of an avatar has benefits for users with respect to identity, learning, inclusion, and engagement [20-23]. Because users often perceive avatars as extensions of themselves, this can enhance their sense of presence, embodiment, and attachment [24]. Also, modifying avatar features to resemble the user improved embodiment, even in healthy populations [25].

Prior studies show that strong embodiment can occur even with dissimilar or stylized avatars as long as key conditions like visuomotor synchrony and first-person perspective are met [4,26-28]. Also, human familiarity with mirrors from early development supports the perception of mirrored avatars as self-representations [29,30]. Therefore, virtual mirrors can further enhance embodiment by allowing users to observe their avatars in motion, including facial expressions [31,32]. Apart from UX benefits, virtual mirrors influence psychological and behavioral outcomes in VR. The “Proteus effect” suggests that avatars can alter user behavior and attitudes in virtual settings [29,33]. However, the virtual mirror potential in VR therapy is unclear and remains underexplored.

Self-compassion is the ability to “soothe oneself with kindness and nonjudgmental understandings in times of difficulty” [34] and is the foundation of compassion-focused therapy (CFT) [35]. In VR therapy, avatars replace human actors to deliver CFT [1,12]. However, there are few established avatar design standards, which may disadvantage neurodivergent populations, such as those with depression, who may require accessible VR design considerations.

Although prior work has demonstrated the therapeutic value of avatar-based CFT in VR, it is unclear whether these benefits generalize to design factors such as avatar stylization, customization, and the use of virtual mirrors. Personalization has been linked to improved body ownership, agency, and immersion [25], while virtual mirrors have shown potential therapeutic benefits in VR counseling [31,32]. These findings motivate the current study, which investigates how avatar appearance and environmental design influence UX and self-compassion in VR therapy. Accordingly, we address two research questions:

- RQ1: Do avatar and environmental design features influence UX?
- RQ2: Do these features influence therapeutic benefit, measured as self-rated self-compassion?

The aim of this study is to examine whether avatar appearance, avatar customization, and the use of virtual mirrors influence UX and self-compassion in VR therapy. We conducted three studies to examine the effects of (1) stylized avatars, (2) customizable stylized avatars, and (3) customizable stylized avatars with a virtual mirror. Each study built upon prior work [3], focusing only on the specified experimental features. A total of 107 neurotypical participants were recruited.

## Methods

### Overview

This manuscript adheres to the STROBE (Strengthening the Reporting of Observational Studies in Epidemiology) guidelines. This study replicates and extends a prior VR-based self-compassion therapy [3], which serves as the control condition for study 1. The original iVR experience included an onboarding interface where participants selected avatars, environments, and companion avatars with emotional behaviors (crying or upset). The therapy consisted of two stages: delivering compassion and receiving compassion.

Three between-subject studies were conducted:

- Study 1: Manipulated avatar appearance
- Study 2: Introduced stylized avatar customization
- Study 3: Introduced a virtual mirror

Each study built on the previous, allowing for pairwise comparisons. Stylized avatars and new features were integrated by editing the original Unity project files.

### Avatar Fidelity

**Figure 1.**
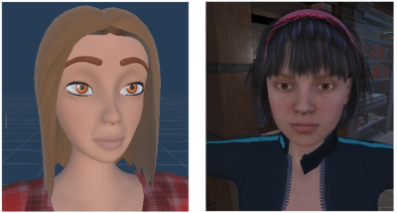
Comparison of stylized and realistic avatars used in the experimental and control conditions of study 1. The stylized avatar (left) featured simplified textures and nonhuman proportions, while the realistic avatar (right) reflected detailed human morphology. Participants (n=20) completed a between-subject virtual reality self-compassion protocol targeting depressive symptoms at The University of Queensland extended reality laboratory (2023‐2024).

Figure 1 compares the stylized avatar used in experimental conditions with the realistic avatar from the original system. Based on Weidner et al [36], stylized avatars feature simplified textures and nonhuman proportions, while realistic avatars maintain human morphology and detail. The avatars of the control study (Figure 1, right) had such realistic features and therefore aligned with generic realistic avatars as defined in Weidner et al [36].

For clarity, for the remainder of the paper, the term “realistic” refers to the generic realistic category [36].

### Measures

Two key instruments were used:

- User Experience Questionnaire (UEQ): Assesses UX across six scales.
- Self-Compassion Scale (SCS) [37]: Measures self-compassion

From study 2 onward, the 8-item Patient Health Questionnaire (PHQ-8) [38], a validated measure for screening depression symptoms, was introduced to support downstream clinical effects of the iVR intervention. As this paper continues the work of Halim et al [3], it was considered prudent to include ongoing depression measurement.

Within study 3, presence was measured using the Slater-Usoh-Steed (SUS) questionnaire [8,39], a 6-item scale to support the analysis of UX. With respect to measurement, presence was prioritized over embodiment measures due to practical constraints; also, as introduced earlier, presence performance implies embodiment quality [13,14].

A qualitative questionnaire gathered open-ended feedback about the avatars (studies 1 and 2) and a virtual mirror (study 3). These questions targeted participants’ likes and dislikes to probe for system improvements and aimed to elicit uncanny valley (UV) responses via an open-ended question in studies 1 and 2:

- What were the top three things that you liked about individualized VR?
- How do you think the next version can be improved?
- How do you feel about your experience with the avatars? (Only in studies 1 and 2.)

In study 3, participants were given questions pertaining to their experience with the virtual mirror:

- How do you feel about your experience with the mirror; what aspects of the mirror did you like or dislike? (Only in study 3.)

In the absence of eye tracking technology, these responses provided a means of validating mirror interactions. Thematic analysis was used to evaluate these responses [40].

### Data Analysis

Two comparisons were made:

- Within-study: pre-post changes in SCS, PHQ-8, and UEQ
- Between-studies: differences across conditions at session completion

For each quantitative data set, the Shapiro-Wilk normality test was first applied. Given the normality of the data, either a Mann-Whitney *U* test or a 2-tailed *t* test was performed. The null hypothesis assumed no significant differences between conditions.

### Onboarding Procedure

#### iVR Therapy Stage 0: Individualization

**Figure 2.**
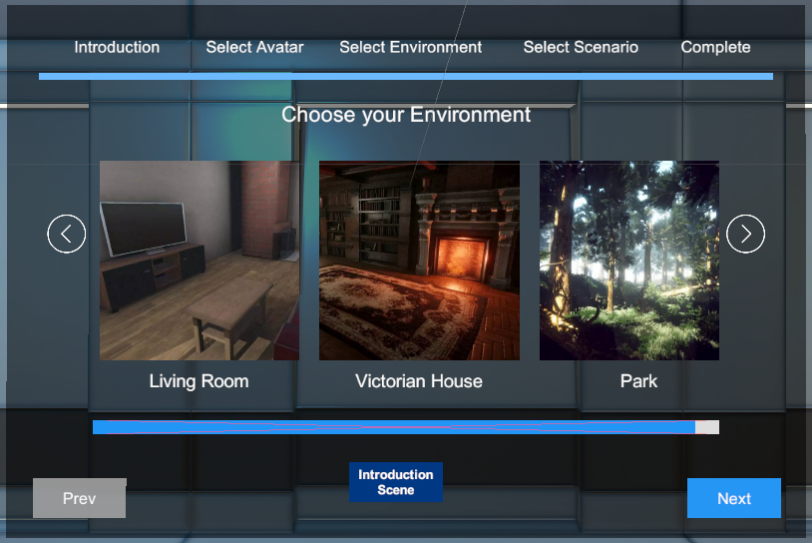
Environment selection interface during the individualization stage of the individualized virtual reality self-compassion therapy. Participants (n=49) in study 2 selected a preferred virtual setting—living room, Victorian house, or park—as part of the avatar and scene customization process. The study targeted depressive symptoms and was conducted at The University of Queensland’s extended reality laboratory (2023‐2024).

From a lobby environment, the participants began by selecting an avatar that resembled themselves, selecting a therapeutic environment (Figure 2), a companion avatar, and the avatar’s behavior. During customization (study 2), participants created avatars that resembled themselves. Movement in the VR scene was fixed to maintain task focus.

#### iVR Therapy Stage 1: Giving Compassion

Participants interacted with their selected companion, who acted out their emotional state before the user. An instruction panel guided the participants through three compassion strategies [1]:

- Validation: Acknowledge and accept the companion’s distress
- Redirection of attention: Shift attention to something more positive
- Memory activation: Encourage recalling a comforting memory (eg, someone who is kind to them)

#### iVR Therapy Stage 2: Receiving Compassion

Participants reentered the same environment from the companion’s perspective and observed their avatar delivering the recorded compassionate message from stage 1. This playback is intended to evoke a therapeutic experience and aligns with prior iVR and VR compassion interventions [3,12]. A second session occurred for participants in studies 2 and 3; these second sessions occurred 2 weeks after initial exposure to the intervention.

### Experimental Procedure

#### Study 1: Stylized Avatars

To explore RQ1 and RQ2, study 1 replaced all avatars with stylized avatars purchased from SunBox Games [41]. Prior work suggests that highly realistic avatars do not necessarily enhance UX in immersive, stressful environments [42,43]. The virtual environment in this study has a unique human-avatar interaction in stage 2, where participants receive compassion from a virtual self—an essential component of self-compassion therapy.

According to Gisbergen et al [42], stylized avatars can potentially avoid UV effects [44], which may arise when avatars do not achieve full human realism. Therefore, by using lower-fidelity avatars, this study aimed to reduce user expectations for realism and improve predictability—key aspects of system dependability as measured by the UEQ. Although dependability is not a direct measure of UV, it serves as a relevant proxy in the context of UV evaluation.

A qualitative method was included to capture UV-related responses, similar to Becker-Asano et al [45]. UEQ and SCS were used for cross-study comparison. Figure 2 shows the avatars for participant selection via the lobby interface.

#### Study 2: Stylized Avatar Customization

**Figure 3.**
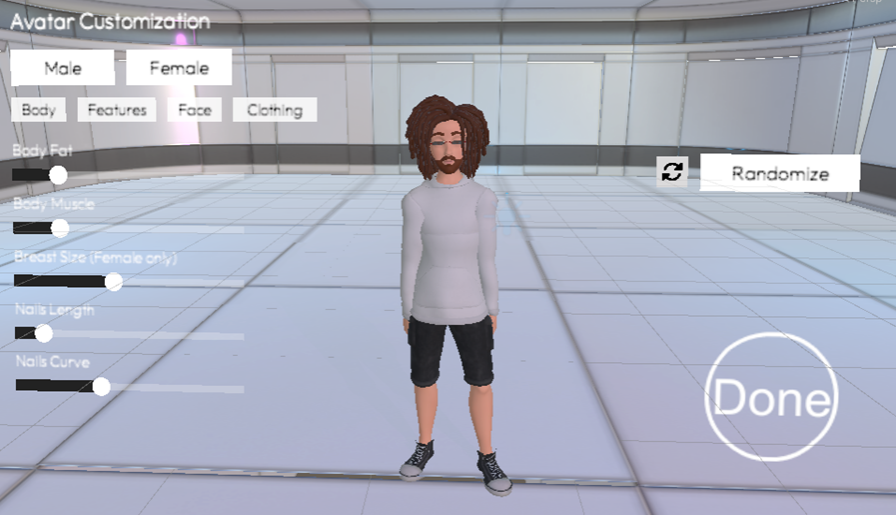
Enhanced avatar customization interface used in study 2 of the virtual reality self-compassion therapy protocol. Participants (n=49) modified a stylized avatar by adjusting body shape, facial features, and clothing using predefined editable parameters. Customization was mandatory and guided by the instruction to create an avatar resembling their self-image. The study targeted depressive symptoms and was conducted at The University of Queensland’s extended reality laboratory (2023‐2024).

Study 2 extended study 1 by adding customization options to stylized avatars (Figure 3). Participants adjusted the avatar using the following SunBox integrated tools [41]:

- Body: 8 parameters—height, fat, muscle, skin, nails, eye, eyelashes, and brow
- Features: hair and facial hair
- Face: 22 facial features (eg, ears, nose, mouth, chin)
- Clothing: 5 items—glasses, hat, tops (shirts, jumpers, etc), bottoms (pants, skirts, etc), and shoes

Customization was required before proceeding. As a quality check, the facilitator encouraged participants to create avatars that resembled themselves within the limitations of the stylized appearance. Participants could not continue unless this criterion was satisfied by a visual check from the researcher supervising. While no formal metric was used, key features such as hairstyle, skin tone, and body shape were considered. All participants engaged fully in the customization process, and informal observation confirmed that stylized avatars generally resembled participants before continuing. Study 2 also added a second iVR session 2 weeks after the first, as this was required by the introduction of the PHQ-8.

#### Study 3: Introduction of Virtual Mirror

**Figure 4.**
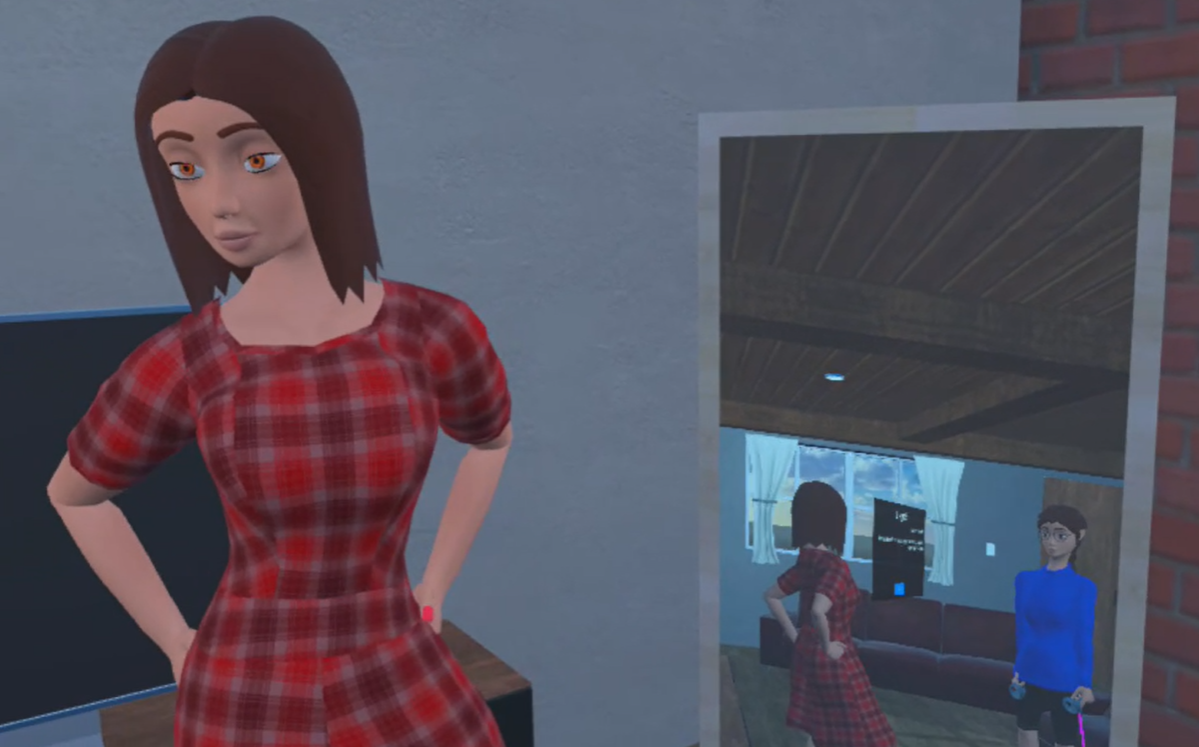
Virtual mirror setup introduced in study 3 of the individualized virtual reality self-compassion therapy protocol. Participants (n=38) viewed real-time reflections of their customized stylized avatars through a virtual mirror positioned to their side during the interaction task. The setup was designed to enhance embodiment and therapeutic presence. The study targeted depressive symptoms and was conducted at The University of Queensland’s extended reality laboratory (2023‐2024). The participant shown is not identifiable and provided consent for inclusion.

Study 3 extended study 2 by introducing a virtual mirror positioned to the side of the user during stage 1 (Figure 4). This design was intended to enhance embodiment and presence, as theorized in the Background subsection, in an effort to elicit improvements in UX and therapeutic outcomes using the current customized stylized avatar format. Participants observed real-time reflections of their avatars without interfering with the primary task.

### Participant and Study Setting

Participants were recruited from the general community via posters, social media, university mailing lists, and word of mouth. Most were students or staff at The University of Queensland. Eligibility required being ≥18 years of age and capable of using immersive VR; those with severe motion sickness or conditions that might interfere with VR use were excluded.

Sessions were conducted one-on-one in a controlled room at The University of Queensland’s extended reality laboratory (Brisbane, Australia), with a trained researcher present. Participants were recruited and tested between 2023 and 2024. Studies 2 and 3 included a 2-week in-person follow-up. Some individuals who registered did not attend their session and were not awarded course credit; no reasons were collected.

Sample sizes were determined pragmatically based on prior VR studies, available resources, and recruitment feasibility in a university setting. No formal power analysis was conducted due to the exploratory nature of the research.

### Ethical Considerations

This study was approved by The University of Queensland Human Research Ethics Committee (approval #2023/HE000468). All participants provided written informed consent prior to participation. Data were de-identified before analysis to protect participant privacy and confidentiality. Participants received course credit through the university’s research participation system. No images or materials in the manuscript or supplementary files contain identifiable individuals.

## Results

### Assessment Schedule

**Figure 5.**
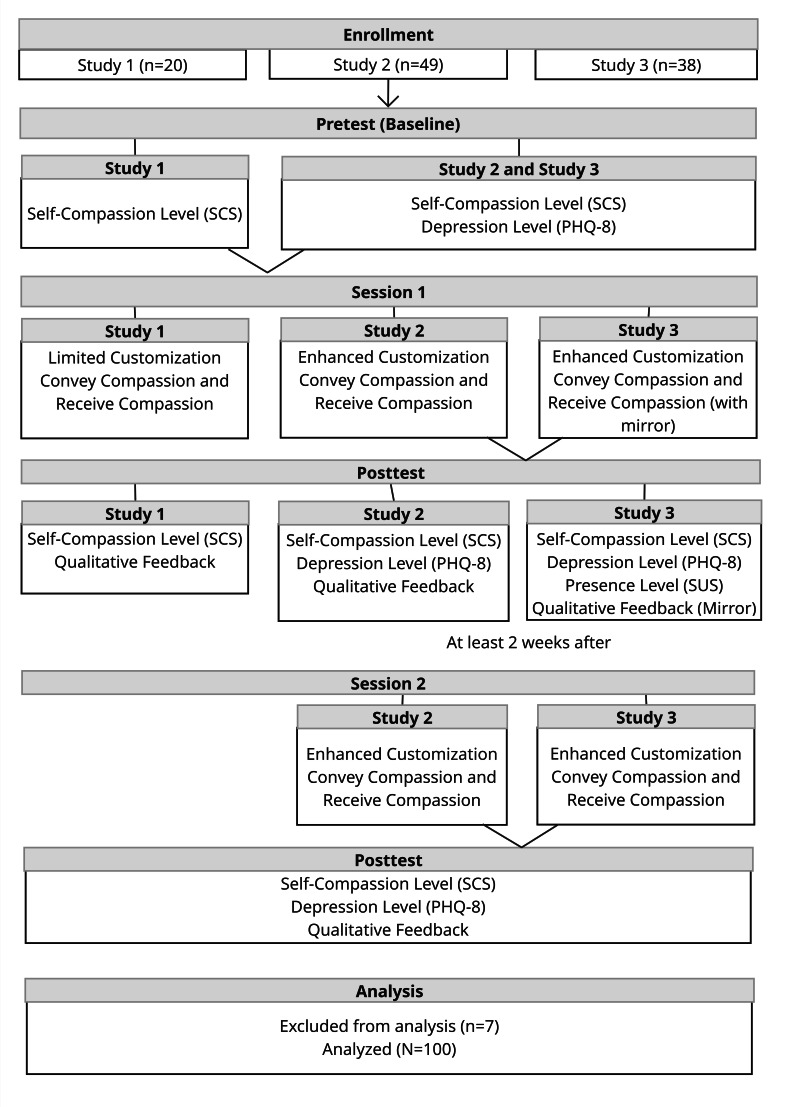
Flow diagram showing participant progression and assessment schedule across three between-subjects studies conducted at The University of Queensland extended reality laboratory (2023‐2024). Participants (N=107) were enrolled in study 1 (n=20), study 2 (n=49), or study 3 (n=38), and 7 were later excluded from the analysis. The studies targeted depressive symptoms and included pre- and posttest measures of self-compassion (SCS), depression (PHQ-8), presence (SUS), and qualitative feedback. Studies 2 and 3 included a second session at least 2 weeks later. Interventions varied by avatar customization and the presence of a mirror. PHQ-8: 8-item Patient Health Questionnaire; SCS: Self-Compassion Scale; SUS: Slater-Usoh-Steed.

A flow diagram showing participant progression and the assessment schedule is presented in Figure 5.

### Study 1: Stylized Avatars

A total of 20 participants completed session 1 (n=12 female participants, n=6 male participants, and n=2 undisclosed).

#### Impact on User Experience (UEQ)

**Figure 6.**
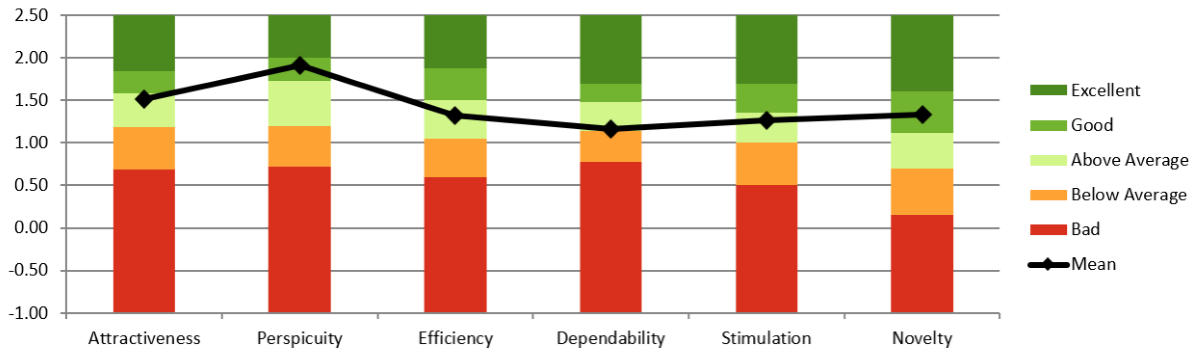
User Experience Questionnaire (UEQ) results from study 1, where participants (n=20; n=12 female, n=6 male, and n=2 undisclosed) interacted with stylized avatars during a single-session virtual reality self-compassion therapy targeting depressive symptoms. Participants completed the UEQ after session 1. Compared to the baseline results using realistic avatars (Figure 7), perspicuity scores improved while other dimensions remained at or above average. The study was conducted at The University of Queensland’s extended reality laboratory (2023).

**Figure 7.**
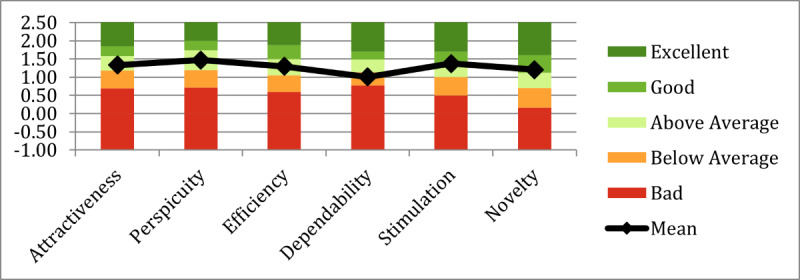
User Experience Questionnaire (UEQ) results from a prior study [3] using realistic avatars in a self-compassion virtual reality therapy protocol. This figure presents participant scores across six UEQ dimensions and serves as a baseline for evaluating the impact of avatar visual style in the current research. The original study targeted depressive symptoms and was conducted at The University of Queensland’s extended reality laboratory (2022). Participants were neurotypical adults recruited from the university community.

At the time of analysis, the UEQ benchmark included 21,175 users from 468 studies assessing software and digital products [46]. As shown in Figure 6, stylized avatars resulted in a category increase in perspicuity compared to the original study, as seen in Figure 7. All UEQ categories remained at least “above average,” indicating no negative impact on the UX. Thus, in relation to RQ1, stylized avatars appear appropriate.

#### Impact of Self-Compassion (SCS)

The mean SCS score in study 1 was 3.11 (SD 1.14; Table S1 in Multimedia Appendix 1). Scores below 2.4 are low, between 2.4 and 3.6 are average, and above 3.6 are high [47]. Therefore, after one VR therapy session, participants reported average levels of self-compassion.

A Shapiro-Wilk test for normality confirmed that data were normally distributed for study 1. A between-studies comparison with Halim et al [3] using a 2-tailed *t* test found no significant difference in SCS between the control group (mean 3.07, SD 0.73) and study 1 (mean 3.11, SD 1.14; t_53_=0.14; *P*=.89; Cohen *d*=0.04). These results indicate stylized avatars had no measurable impact on self-compassion (RQ2).

### Study 2: Stylized Avatar Customization

A total of 49 participants completed session 1; 42 (86%) returned for session 2. The gender of the participants was as follows:

- Session 1: 22 female, 22 male, 5 undisclosed
- Session 2: 18 female, 21 male, 3 undisclosed

#### Impact on User Experience (UEQ)

**Figure 8.**
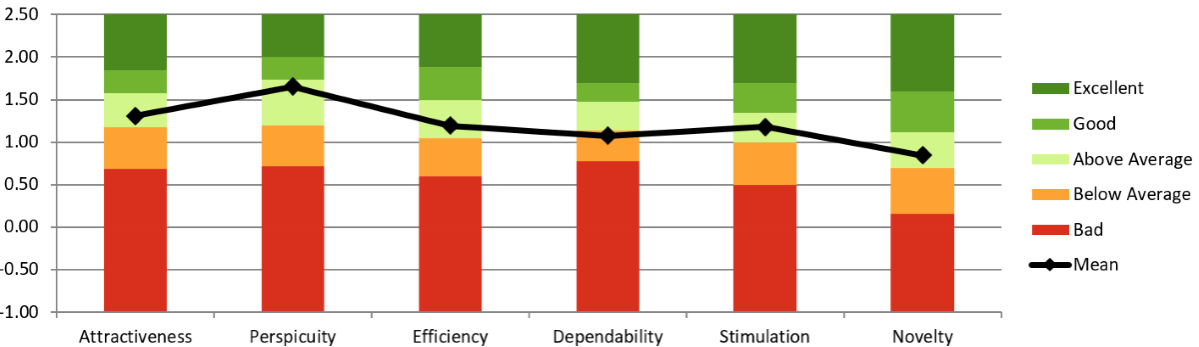
User Experience Questionnaire (UEQ) results from study 2 (n=49; session 1), in which participants interacted with personalized stylized avatars during a virtual reality self-compassion therapy targeting depressive symptoms. Participants completed the UEQ after their first session. Compared to study 1, mean scores decreased in perspicuity, dependability, and novelty, with only dependability rated below the neutral benchmark. The study was conducted at The University of Queensland’s extended reality laboratory (2023‐2024).

Study 2 reused the same UEQ tool as study 1. As seen in Figure 8, scores declined in perspicuity, dependability, and novelty, with only dependability dropping below average. Given a between-studies comparison (Table S2 in Multimedia Appendix 1), UX differences were not statistically significant; therefore, with respect to RQ1, we did not find evidence that stylized avatar customization significantly influenced UX under the conditions tested.

**Figure 9.**
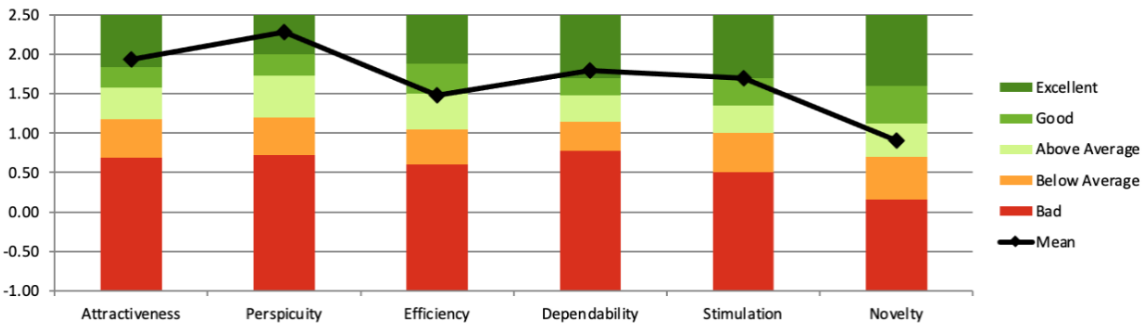
User Experience Questionnaire (UEQ) results from study 3 (n=38; session 1), where participants engaged in virtual reality self-compassion therapy using stylized avatars and a virtual mirror. Compared to study 2, scores significantly improved in attractiveness, perspicuity, dependability, and stimulation, with three dimensions rated as “excellent.” Assessments were completed after the first session. The study was conducted at The University of Queensland’s extended reality laboratory (2023‐2024) and targeted depressive symptoms.

#### Impact of Self-Compassion (SCS)

For a within-study comparison, the Shapiro-Wilk test for normality confirmed that data were normally distributed. As seen in Table 1, a 2-tailed *t* test comparing baseline and session 2 SCS in study 2 revealed a statistically significant difference with a moderate effect size. These results provide evidence that customization positively influenced the therapeutic outcome (RQ2).

**Table 1.** Summary statistics and internal comparisons from study 2 of a three-part mixed methods study investigating virtual reality self-compassion therapy for depressive symptoms.^a^

Measures	Mean (SD) or mean rank	Mann-Whitney *U* test	*t* test (df) or *z*	*P* value (2-tailed)	Effect size (Cohen *d* or *r*)
UEQ^b^		—^c^	—	—	—
Attractiveness	1.31 (0.85)				
Perspicuity	1.65 (0.96)				
Efficiency	1.19 (0.79)				
Dependability	1.07 (0.81)				
Stimulation	1.18 (0.96)				
Novelty	0.85 (1.01)				
SCS^d^					
Baseline	3.05 (0.98)	—	—	—	—
Session 2	3.55 (1.16)	—	—	—	—
Comparison	—	—	2.219 (89)^e^	.03	−0.47
PHQ-8^f^					
Baseline	46.16^g^	—	—	—	—
Session 2	44.71^g^	—	—	—	—
Comparison	—	972	0.26^h^	.79	0.02^i^

a Participants (n=49 at session 1; n=42 at session 2) interacted with personalized stylized avatars. Measures included the UEQ, SCS, and PHQ-8. The study was conducted at The University of Queensland extended reality laboratory between 2023 and 2024 using a between-subjects design.

b UEQ: User Experience Questionnaire.

c Not applicable

d SCS: Self-Compassion Scale.

e *t* test value.

f PHQ-8: 8-item Patient Health Questionnaire.

g Mean rank value.

h *z* value.

i *r* value.

#### Impact on Depression (PHQ-8)

The Shapiro-Wilk test for normality revealed nonnormal distribution; thus, a Mann-Whitney *U* test was used. No significant difference was found between baseline and session 2 PHQ-8 scores, with a negligible effect size (see Table 1).

### Study 3: Introduction of Virtual Mirror

A total of 38 participants completed session 1; 35 (92%) returned for Session 2. Gender of the participants was as follows:

- Session 1: 20 female, 18 male
- Session 2: 20 female, 15 male

#### Impact on User Experience (UEQ)

No changes to the UEQ tool have been made since study 2. As seen in Figures8  9, study 3 scored higher in attractiveness, perspicuity, dependability, and stimulation compared to study 2. Three of six dimensions were rated “Excellent.” Given a between-studies comparison (Table S2 in Multimedia Appendix 1), UX differences were statistically significant; therefore, we found evidence that stylized avatar customization and virtual mirrors significantly influenced UX. These findings support RQ1, suggesting that stylized avatar customization and virtual mirrors significantly influenced UX.

#### Impact of Self-Compassion (SCS)

After removing five participants who did not complete the full SCS questionnaire, the Shapiro-Wilk test confirmed that data were normally distributed. A within-study, 2-tailed *t* test revealed no significant difference between baseline and session 2 SCS, with a small effect size (see Table 2). These results do not provide evidence that mirrors influenced self-compassion outcomes (RQ2).

**Table 2.** Summary statistics and internal comparisons from study 3 of a three-part mixed methods investigation into virtual reality self-compassion therapy for depressive symptoms.^a^

Measures	Mean (SD) or mean rank	Mann-Whitney *U* test	*t* test or *z*	*P* value (2-tailed)	Effect Size (Cohen *d* or *r*)
UEQ^b^		—^c^	—	—	—
Attractiveness	1.88 (0.92)				
Perspicuity	2.22 (0.65)				
Efficiency	1.42 (1.04)				
Dependability	1.76 (0.75)				
Stimulation	1.65 (1.02)				
Novelty	0.87 (1.04)				
SCS^d^					
Baseline	3.88 (1.33)	—	—	—	—
Session 2	4.09 (1.05)	—	—	—	—
Comparison	—	—	0.71 (63)^e^	.47	0.18
PHQ-8^f^					
Baseline	44.93^g^	—	—	—	—
Session 2	32.07^g^	—	—	—	—
Comparison	—	477.5	2.53^h^	.01	0.30^i^

a Participants (n=38 at session 1; n=35 at session 2) interacted with personalized stylized avatars and received visual feedback via a virtual mirror. Measures included the UEQ, SCS, and PHQ-8. The study was conducted at The University of Queensland extended reality laboratory between 2023 and 2024 using a between-subjects design.

b UEQ: User Experience Questionnaire.

c Not applicable.

d SCS: Self-Compassion Scale.

e *t* test value.

f PHQ-8: 8-item Patient Health Questionnaire.

g Mean rank value.

h *z* value.

i *r* value.

#### Impact on Depression (PHQ-8)

The data were not normally distributed; therefore, a Mann-Whitney *U* test found a significant reduction in PHQ-8 scores from baseline to session 2 (see Table 2). This result suggests a potential therapeutic benefit associated with the inclusion of stylized avatar customization and virtual mirrors.

#### Impact on Presence (SUS)

The mean SUS score in study 3 (mean 4.56, SD 1.58, 95% CI 4.02-5.10) overlapped with the benchmark SUS scores in Usoh et al [39], which reported a virtual (mean 3.8) and a real (mean 4.4) environment. This suggests that presence in our virtual mirror condition fell within a comparable range previously validated by Usoh et al[39] and did not significantly differ from real-world experience (*t*_46_=0.30; *P*=.77; *d*=0.18). The lower bound of our CI exceeded the virtual benchmark, suggesting a relatively strong presence in the mirror condition.

### Cross-Study Qualitative Analysis

Open-ended feedback was collected from 100 participants across all three studies; participants completed their assigned iVR therapy exposures prior to providing responses. Deductive and inductive thematic analysis was conducted on participants’ open-ended responses. Participants were labeled P1-P20 (study 1), P21-P62 (study 2), and P63-P100 (study 3). The deductive thematic approach (Table S3 in Multimedia Appendix 1) includes codes corresponding to UEQ dimensions from Schrepp et al [46], which provided a structured lens through which to interpret qualitative feedback.

In parallel, an inductive analysis was performed to identify emergent themes not captured by the UEQ framework. These data-driven themes reflected patterns in participant experience that appeared to be consistent with experimental changes. The results of the inductive analysis are summarized in Table S4 in Multimedia Appendix 1.

## Discussion

### Overview

This study explored how stylized avatars, avatar customization, and virtual mirrors influenced UX (RQ1) and self-compassion outcomes (RQ2) across three iVR therapy conditions. In study 1, qualitative feedback indicated general acceptance of the stylized avatars. Study 2 showed a significant improvement in self-compassion outcomes compared to study 1. Study 3 produced significant improvements in four of the six UEQ dimensions, which suggests improved UX.

### Summary of User Experience (RQ1)

For cross-study comparisons, see Table S2 in Multimedia Appendix 1. Study 3 yielded the highest attractiveness scores, with a statistically significant improvement compared to study 2 (*P*=.01). Qualitative data indicated this may be attributed to the enjoyment of avatar customization, which was introduced in study 2 and praised by participants in study 3. However, immersion-related feedback also emerged, such as “some of the environment designs feel too rigid…which breaks the immersion” (P78). Since Study 3 introduced virtual mirrors—known to support embodiment—this addition may explain the rise in UX.

On the perspicuity scale, study 3 again scored highest and significantly outperformed study 2 (*P*=.01). Participants highlighted the ease of learning, but across all studies, a lack of narrative context was noted. For example, participants reported difficulty understanding the avatars’ emotional states, affecting their ability to engage in role-play scenarios.

Study 3 also significantly improved dependability scores (*P*<.001). The stylized avatars, rather than triggering discomfort, were perceived as appealing—“they’re all cute” (P37)—and met participant expectations for a supportive environment. Thematic analysis suggested that the participants felt the system supported therapeutic outcomes: “I think this can make me feel relaxed” (P83).

Stimulation was significantly higher in study 3 than in study 2 (*P*=.04). Participants reported increased interest when viewing themselves delivering compassion, suggesting that the mirror feature enhanced engagement.

With respect to novelty, all studies received positive feedback—“innovative and unique” (P20)—but only study 1 reached an “above average” benchmark. Some participants reported reduced novelty due to restricted avatar movement, for example, “better if the users got a little more freedom during the simulation” (P56). This may indicate a design trade-off between structured therapeutic focus and exploratory freedom.

### Interpreting User Experience

The UEQ served as the primary UX tool due to its alignment with iVR design goals for mental health. Though no significant differences were observed between studies 1 and 2, stylized avatars performed well compared to the UEQ benchmark, validating their continued exploration in therapeutic contexts. However, despite enhanced customization in study 2, UEQ scores did not improve significantly. One possible explanation may lie in elevated participant expectations (eg, limited skin tone options), which may have led to reduced satisfaction. This supports the idea that “less is more”; a limited but well-executed customization system may better serve therapeutic VR experiences.

Feedback across studies emphasized the importance of avatar individualization for user acceptance. Study 3’s improvement in multiple UEQ dimensions suggests that mirrors—without being explicitly highlighted—supported user presence (and likely embodiment). “Attractiveness is a pure valence dimension” [46], that is, representative of the user’s general experience. Attractiveness rose significantly in study 3, likely due to parallel improvements in perspicuity, dependability, and stimulation.

This connection is further supported by SUS findings. Significant improvements on items related to perceived realness—questions 2 and 3 of the SUS questionnaire [8]—and memory of the experience indicate that participants recalled the virtual mirror scenario as immersive and believable. Given the high scores (Table S5 in Multimedia Appendix 1), this kind of recall implies a strong sense of presence [39].

Taken together, study 3 suggests that the inclusion of virtual mirrors was a key turning point in enhancing iVR UX. While prior studies did not yield significant gains, mirrors may have “unlocked” the potential of the design. Whether these effects are solely due to mirrors or the cumulative impact of prior changes remains unclear, but no prior condition showed similar improvements.

### Summary of Therapeutic Outcomes (RQ2)

In study 2, participants exposed to customizable stylized avatars over two sessions showed a significant increase in self-compassion (SCS). This supports the notion that stylistic, customized avatars in iVR interactions may enhance therapeutic benefit. However, these gains were not observed in study 3, despite the enhanced UX. This suggests that while mirrors improve UX and presence, they may not directly translate to better therapeutic outcomes.

Nevertheless, qualitative feedback highlighted that participants valued the ability to create avatars resembling themselves. It was noted that this customization improved their emotional connection to the experience in stage 2 of the experiment. For example, “customizing it to look like me did make the difference of how I perceived the audio replay” (P60). This statement implies that appearance alone was insufficient; users needed a visual feedback loop (eg, mirrors) to fully benefit from avatar customization.

Interestingly, mirror placement was not emphasized to participants yet still yielded a significant impact. However, incorrect or unnatural mirror placement disrupted immersion. For example, “[mirror] could be weird [to see] in the park environment” (P64) or “I see the mirror and then walk through it. I’m [a] ghost and that makes me a little bit scar[ed]” (P76). This underscores the importance of environmental congruence in design-embodied iVR therapy.

### The Uncanny Valley

The appearance of stylized avatars was generally well received, supporting the idea that less realistic avatars can be effectively used in emotionally sensitive and immersive environments such as iVR self-compassion therapy, where avatar-owner resemblance is important. This finding aligns with prior research suggesting that stylized avatars may reduce the risk of UV effects while still fostering user connection [42,43,48]. Participants responded positively to avatars with cartoonish or humanoid qualities. “I don’t recommend realistic avatars as much. I think the more cartoonish appeal is better” (P41).

The UV model suggests that entities close to human-likeness [44], but imperfect, can cause discomfort. The use of stylized avatars in this study—located near “Humanoid Robot” on the affinity line—appears to have mitigated this risk. Mirrors further supported predictability and safety (dependability), suggesting that this combination may serve as a UX-enhancing strategy to avoid UV interference.

That said, UV was not the primary aim of this study but a theoretical background for design rationale. However, these reflections are offered as considerations for future iVR therapy design. It is important to note that violations of physical logic (eg, walking through mirrors, unnatural placement) also negatively impacted immersion and should be carefully addressed in future iterations.

### Implications and Design Reflections

This study shows that stylized avatars, when paired with suitable UX enhancements such as virtual mirrors and avatar customization (along with other individualization concepts, environment selection, companion avatars, emotional behavior, etc), can offer stronger UX without losing therapeutic benefit, which might be an assumed consequence of selecting a lesser fidelity avatar. Pursuing hyperrealistic avatars without considering context may be counterproductive, as this ignores the Sisyphus-like limitation that avatars will risk falling back into the UV when realism is applied without careful design intent. This study gathers evidence to suggest that lower-fidelity avatars can be more appropriate in therapeutic settings but also that context matters when selecting avatar designs.

### Limitation

This study used the SCS—a validated tool—but it has known psychometric limitations [49]. Additionally, participants were not filtered based on PHQ-8 scores; however, 74% had mild or higher range depression results. Future study iterations should include codesign and testing with clinical populations.

While the fidelity comparison was carefully controlled across studies, data were collected at different time points, which introduces the possibility of unmeasured societal or contextual differences. Future studies should aim to run control and experimental conditions concurrently to further minimize potential time-related confounds. Also, avatar fidelity was classified using a binary framework [36]. Future research may benefit from applying more continuous fidelity measures—such as user-rated realism scales or quantitative visual complexity metrics—to better capture subtle design differences.

Mirror engagement was inferred rather than directly measured, as the Meta Quest 2 headset lacks built-in eye tracking. However, mirrors were centrally positioned, and qualitative responses strongly suggest that participants did engage with them.

Finally, some tests were underpowered, which limited the ability to detect small-to-moderate effects. To help interpret these findings and provide additional insight, qualitative methods were included to support quantitative results.

### Conclusion

If compassion means “to suffer together” [50], then does engaging in self-compassion mean suffering alone? Perhaps not with these avatars. In this iVR intervention, participants were comforted by their own stylized, customized avatars within immersive environments enhanced by virtual mirrors. Participants experienced self-compassion in a space tailored to their preferences, offering new insights into how avatar design and environmental cues shape therapeutic experiences.

This study sequentially investigated whether stylized avatars, avatar customization, and virtual mirrors influence UX and therapeutic outcomes in self-compassion VR therapy. Across three studies, results demonstrated that stylized avatars were well accepted and did not diminish self-compassion outcomes. Avatar customization improved self-compassion scores in one study, while virtual mirrors significantly improved UX across multiple dimensions. However, enhanced UX alone did not guarantee stronger therapeutic outcomes.

These findings suggest that individualized avatars and virtual mirrors can meaningfully enhance iVR experiences without triggering UV effects. More broadly, this study supports the feasibility of lower-fidelity avatars in mental health VR applications. Future research should explore these design strategies in clinical populations to better understand avatar-based therapy interventions.

## Supplementary material

10.2196/71004 Multimedia Appendix 1 Tables presenting additional quantitative and qualitative outcomes across studies 1-3.

## Abbreviations

CFT: compassion-focused therapy
iVR: individualized virtual reality
PHQ-8: 8-item Patient Health Questionnaire
SCS: Self-Compassion Scale
STROBE: Strengthening the Reporting of Observational Studies in Epidemiology
SUS: Slater-Usoh-Steed
UEQ: User Experience Questionnaire
UV: uncanny valley
UX: user experience
VR: virtual reality
