# Supplementary material for: Avatar Customization and Embodiment in Virtual Reality Self-Compassion Therapy for Depressive Symptoms: Three-Part Mixed Methods Study
Source: JMIR Form Res. 2025 Oct 2;9:e71004. doi: 10.2196/71004 (PMC12490737; doi:10.2196/71004)
Supplement: Multimedia Appendix 1 [file formative-v9-e71004-s001.docx]

Table S1. Summary Statistics for Study 1: Stylized Avatars in VR Self-Compassion Therapy. Participants (n = 20) completed a single-session immersive VR self-compassion intervention using stylized avatars at The University of Queensland XR Lab (2023). Outcomes included the Self-Compassion Scale (SCS) and User Experience Questionnaire (UEQ). Reported values include means and standard deviations for each subscale.

|  | UEQ Scale | Mean (SD) |
| --- | --- | --- |
|  |  |  |
| **SCS** |  |  |
|  |  | 3.11 (1.14) |
| **UEQ** |  |  |
|  | Attractiveness | 1.51 (0.97) |
|  | Perspicuity | 1.91 (1.33) |
|  | Efficiency | 1.32 (1.11) |
|  | Dependability | 1.17 (0.86) |
|  | Stimulation | 1.26 (1.13) |
|  | Novelty | 1.33 (0.97) |

Table S2. Between-Study Comparisons of UEQ Scores Across Three VR Studies (2023–2024). Mann-Whitney U tests compared User Experience Questionnaire scores across participants in Study 1 (n = 18), Study 2 (n = 47), and Study 3 (n = 34), investigating the impact of avatar customization and virtual mirror interventions in a VR therapy context for depressive symptoms. Reported metrics include mean ranks, U values, z-scores, p-values, and effect sizes.

|  |  | Mean Rank | Mann-Whitney U | z | P value (2-tailed) | Effect Size (r) |
| --- | --- | --- | --- | --- | --- | --- |
|  |  |  |  |  |  |  |
| UEQ Study 1 Vs Study 2: Significance of Stylized Avatar Customization |  |  |  |  |  |  |
| Attractiveness |  |  |  |  |  |  |
|  | Study 1 | 37.69 |  |  |  |  |
|  | Study 2 | 31.20 |  |  |  |  |
|  | Attractiveness Comparison |  | U = 338.5 | 1.24 | .21 | .13 |
| Perspicuity |  |  |  |  |  |  |
|  | Study 1 | 39.14 |  |  |  |  |
|  | Study 2 | 30.65 |  |  |  |  |
|  | Perspicuity Comparison |  | U = 312.5 | 1.63 | .10 | .16 |
| Efficiency |  |  |  |  |  |  |
|  | Study 1 | 37.33 |  |  |  |  |
|  | Study 2 | 31.34 |  |  |  |  |
|  | Efficiency Comparison |  | U = 345.0 | 1.15 | .25 | .12 |
| Dependability |  |  |  |  |  |  |
|  | Study 1 | 38.00 |  |  |  |  |
|  | Study 2 | 31.90 |  |  |  |  |
|  | Dependability Comparison |  | U = 333.0 | 1.33 | .18 | .13 |
| Stimulation |  |  |  |  |  |  |
|  | Study 1 | 34.86 |  |  |  |  |
|  | Study 2 | 32.29 |  |  |  |  |
|  | Stimulation Comparison |  | U = 389.50 | 0.49 | .62 | .05 |
| Novelty |  |  |  |  |  |  |
|  | Study 1 | 39.36 |  |  |  |  |
|  | Study 2 | 30.56 |  |  |  |  |
|  | Novelty Comparison |  | U = 308.50 | 1.69 | .09 | .17 |
|  |  | Mean Rank | Mann-Whitney U | z | P value (2-tailed) |  |
| UEQ Study 2 Vs Study 3: Significance of Virtual Mirrors |  |  |  |  |  |  |
| Attractiveness |  |  |  |  |  |  |
|  | Study 2 | 34.70 |  |  |  |  |
|  | Study 3 | 49.71 |  |  |  |  |
|  | Attractiveness Comparison |  | U = 1095.00 | 2.84 | .01 | .29 |
| Perspicuity |  |  |  |  |  |  |
|  | Study 2 | 34.98 |  |  |  |  |
|  | Study 3 | 49.32 |  |  |  |  |
|  | Perspicuity Comparison |  | U = 1082.00 | 2.72 | .01 | .27 |
| Efficiency |  |  |  |  |  |  |
|  | Study 2 | 37.88 |  |  |  |  |
|  | Study 3 | 45.31 |  |  |  |  |
|  | Efficiency Comparison |  | U = 945.50 | 1.41 | .16 | .14 |
| Dependability |  |  |  |  |  |  |
|  | Study 2 | 32.89 |  |  |  |  |
|  | Study 3 | 52.21 |  |  |  |  |
|  | Dependability Comparison |  | U = 1180.00 | 3.66 | < .001 | .37 |
| Stimulation |  |  |  |  |  |  |
|  | Study 2 | 36.33 |  |  |  |  |
|  | Study 3 | 47.46 |  |  |  |  |
|  | Stimulation Comparison |  | U = 1018.50 | 2.11 | .04 | .21 |
| Novelty |  |  |  |  |  |  |
|  | Study 2 | 41.28 |  |  |  |  |
|  | Study 3 | 40.62 |  |  |  |  |
|  | Novelty Comparison |  | U = 786.00 | 0.13 | .90 | .01 |

Table S3. Deductive Thematic Analysis of Qualitative Feedback Aligned to UEQ Dimensions. Thematic coding of participant responses (Studies 1–3; N = 107) was structured according to the six UEQ dimensions (e.g., Attractiveness, Perspicuity, Efficiency). Representative quotes illustrate participant evaluations of avatar design, emotional experience, and VR system usability.

|  | Theme | Code | Study | Excerpts |
| --- | --- | --- | --- | --- |
|  |  |  |  |  |
| **Attractiveness** |  |  |  |  |
|  | Engagement | Enjoyable | 2 & 3 | "I can design my own character, very cool", "the whole process. I quite like it, and quite creative." and "good experience, it’s my first time to use VR". |
| **Perspicuity** |  |  |  |  |
|  | Intuitive | Easy | All | "Simple and easy to go through" and "easy to follow instructions" |
|  |  | Easy to learn | All | "easy to learn" and "The prompts to help you when trying to calm the person down." |
|  | Experience and Reflection | Easy to learn | 2 | "allowed me to explore different ways that I could use to phrase my words in those situations" |
|  |  | Understandable | 2 & 3 | "The experience taught me that reassurance and not talking about myself" and "each Step is easy to understand" |
|  | Context | Difficult to learn | 1 | "Add some extra contexts pertinent to the user" and "Perhaps there could be a backstory as to why the avatar is upset". |
|  |  | Confusing | All | "Perhaps there could be a backstory as to why the avatar is upset" and "hard to provide compassion when I didn’t know what they were so upset a" |
| **Dependability** |  |  |  |  |
|  | Expectations of Application | Meets Expectations | All | "choice of environment and avatar and realistic crying" and "It was realistic". |
|  | Accuracy | Not Meeting Expectations | 1 & 2 | "I think the degree of personalisation can be improved on", "[more avatar] emotions", and "it would be nice for the avatar to react to our dialogue" |
|  | Uncanny Valley | Secure | 2 | "they’re all cute" |
|  | Therapy Expectations | Meets Expectations | 3 | "I think this can make me feel relaxed" and  "[I] become more happy" |
|  | Virtual Environment | Not Meeting Expectations | 3 | "[scenes] work in a stylised context, but in a [natural] environment, like the park, the immersion breaks". |
| **Stimulation** |  |  |  |  |
|  | Learning Experience | Valuable | 3 | "I see myself, I comfort myself, it really makes [feel] release", "I want to sit on the chair to talk with someone", and "the scenario is very good" |
|  | Avatar Customisation | Interesting | 3 | "Personal[ising] your character [is] very interesting" |
| **Novelty** |  |  |  |  |
|  | Customisation | Creative | 2 & 3 | "the experience allowed for continuous creation and novel experiences through variations", "create your own avatar to look like you, with there being a multitude of options" and "I liked the ability to create a character that could seem like myself" |
|  | New Experience | Innovative | All | "It was new experience" and "each scenario I engaged in was unique and it did slightly alter my interactions" |

Table S4. Inductive Thematic Analysis of User Experience Themes Across All Studies. Qualitative feedback from participants in Studies 1–3 was analyzed thematically to extract emergent themes on avatar individualisation, interaction design, system performance, emotional impact, and mirror reflections. Quotes reflect participant engagement with immersive self-compassion experiences targeting depressive symptoms.

| Theme | Code | Excerpts |
| --- | --- | --- |
|  |  |  |
| Compassion | Therapeutic Benefit | Some participants mentioned that they liked listening to their own voice as it was played back to them via their avatar; “Listening to myself was an interesting experience but it worked” and, “it was nice being able to hear yourself comfort someone else, and I felt I benefited from that”. |
| Avatar Individualisation | Customisation | Study 2 and 3 included a feature which allowed for customisation of the avatar, hence participants stated "I liked the ability to create a character like myself" but also stated "customising it to look like me did make the difference of how I perceived the audio replay" |
| Dialogue Context | Interactions | The participants made requests for avatar responses to help guide their experience; "have some kind of method for response, it’s difficult to comfort a character you can’t interact with”. |
| Avatar Improvement | Avatars | Considering statements about avatar behaviour and appearance, the participants have been quoted as saying “I think the degree of personalisation can be improved” and "options for avatar styles could have more variations (e.g hairstyle, colours, dress types)" and, "there wasn’t a skin colour that matched”. |
| System Issues | Performance | This theme considered matters such as graphics, audio and game mechanics which includes feedback such as; “The graphics could be improved so that it feels less computer-like.”, “More [NPC] actions can be implemented and audio can be synchronized with the body language of the avatar”, and “there were some bugs in the current system, minor, but if that was resolved I reckon the entire user experience would be much smoother”. |
| Self Recognition | Mirror | This theme considered codes such as realisation and influence the participants expressed; "aware [of] what I’m doing [in] that scene." and “[I] see it". |
| Plausibility | Mirror | The mirrors tend to have a bad influence if the placement of the mirror is not consistent with reality. A code named ‘weird in the park’ emerged from the excerpts where participants complained "it’s a bit weird the look at the mirror in the park". |
| Compassion | Therapeutic Benefit | Some participants mentioned that they liked listening to their own voice as it was played back to them via their avatar; “Listening to myself was an interesting experience but it worked” and, “it was nice being able to hear yourself comfort someone else, and I felt I benefited from that”. |
| Avatar Individualisation | Customisation | Study 2 and 3 included a feature which allowed for customisation of the avatar, hence participants stated "I liked the ability to create a character like myself" but also stated "customising it to look like me did make the difference of how I perceived the audio replay" |
| Dialogue Context | Interactions | The participants made requests for avatar responses to help guide their experience; "have some kind of method for response, it’s difficult to comfort a character you can’t interact with”. |
| Avatar Improvement | Avatars | Considering statements about avatar behaviour and appearance, the participants have been quoted as saying “I think the degree of personalisation can be improved” and "options for avatar styles could have more variations (e.g hairstyle, colours, dress types)" and, "there wasn’t a skin colour that matched”. |
| System Issues | Performance | This theme considered matters such as graphics, audio and game mechanics which includes feedback such as; “The graphics could be improved so that it feels less computer-like.”, “More [NPC] actions can be implemented and audio can be synchronized with the body language of the avatar”, and “there were some bugs in the current system, minor, but if that was resolved I reckon the entire user experience would be much smoother”. |
| Self Recognition | Mirror | This theme considered codes such as realisation and influence the participants expressed; "aware [of] what I’m doing [in] that scene." and “[I] see it". |
| Plausibility | Mirror | The mirrors tend to have a bad influence if the placement of the mirror is not consistent with reality. A code named ‘weird in the park’ emerged from the excerpts where participants complained "it’s a bit weird the look at the mirror in the park". |
| Compassion | Therapeutic Benefit | Some participants mentioned that they liked listening to their own voice as it was played back to them via their avatar; “Listening to myself was an interesting experience but it worked” and, “it was nice being able to hear yourself comfort someone else, and I felt I benefited from that”. |
| Avatar Individualisation | Customisation | Study 2 and 3 included a feature which allowed for customisation of the avatar, hence participants stated "I liked the ability to create a character like myself" but also stated "customising it to look like me did make the difference of how I perceived the audio replay" |
| Dialogue Context | Interactions | The participants made requests for avatar responses to help guide their experience; "have some kind of method for response, it’s difficult to comfort a character you can’t interact with”. |

Table S5. Slater-Usoh-Steed (SUS) Presence Scores and Item-Level Comparisons for Study 3 (Virtual Mirror Intervention). Participants (n = 38 at Session 1; n = 35 at Session 2) completed presence assessments after using VR self-compassion therapy with a virtual mirror. Group-level and item-level comparisons are shown for the SUS ‘Virtual’ and ‘Real’ scales. Reported values include means, standard deviations, t-statistics, p-values, and Cohen’s d effect sizes.

|  |  | Mean (SD) | t-test | P value (2-tailed) | Effect Size (Cohen’s d) |
| --- | --- | --- | --- | --- | --- |
| SUS |  |  |  |  |  |
|  | Slater-Usho-Steed ’Virtual’ | 3.8 (1.3) |  |  |  |
|  | Virtual Mirror Intervention | 4.56 (1.58) |  |  |  |
|  | Comparison |  | t46 = 1.57 | .12 | .53 |
|  | Slater-Usho-Steed ’Real’ | 4.4 (1.5) |  |  |  |
|  | Virtual Mirror Intervention | 4.56 (1.58) |  |  |  |
|  | Comparison |  | t46 = 0.30 | .77 | .18 |
|  | Question Analysis | Mean (SD) | t-test | P value (2-tailed) |  |
| SUS Question Breakdown |  |  |  |  |  |
|  | Slater-Usho-Steed ’Virtual’ Q2 | 3.6 (1.3) |  |  |  |
|  | Virtual Mirror Intervention Q2 | 4.63 (1.48) |  |  |  |
|  | Comparison |  | t46 = 2.16 | .04 | .74 |
|  | Slater-Usho-Steed ’Virtual’ Q3 | 2.6 (1.6) |  |  |  |
|  | Virtual Mirror Intervention Q3 | 4.66 (1.56) |  |  |  |
|  | Comparison |  | t46 = 3.64 | < .01 | 1.30 |
